# Primary School Teachers' Knowledge and Misconceptions Regarding Attention Deficit Hyperactivity Disorder in the Qassim Region, Saudi Arabia

**DOI:** 10.7759/cureus.79108

**Published:** 2025-02-16

**Authors:** Abdullah Alqifari, Hana Alqifari, Jood M Alsogaihi, Jolan S Alsaud, Hussam Alshetwi, Mohammed Y Almohaimeed, Shikhah G Alharbi, Shoog S Alhaggas

**Affiliations:** 1 Department of Psychiatry, Qassim University, Buraydah, SAU; 2 Department of Statistics and Operation Research, College of Science, Qassim University, Buraydah, SAU; 3 Medicine, College of Medicine, Qassim University, Buraydah, SAU; 4 Family Medicine, Qassim Health Cluster, Buraydah, SAU

**Keywords:** adhd, attention deficit disorder with hyperactivity, attention-deficit/ hyperactivity disorder, knowledge, therapeutic misconception

## Abstract

Background and objective

Attention deficit hyperactivity disorder (ADHD) is a common neurodevelopmental disorder affecting children's academic and social performance. This study aimed to assess the knowledge and misconceptions of primary school teachers in the Qassim region regarding ADHD.

Methodology

A cross-sectional study was conducted from March 2024 to October 2024 involving 381 primary school teachers in the Qassim region, Saudi Arabia. Data were collected using a self-administered questionnaire, which included demographic information and a 36-item ADHD knowledge assessment. The collected data were analyzed using SPSS Statistics version 25 (IBM Corp., Armonk, NY).

Results

Most participants were female (58.8%, n=224) and aged between 41 and 50 (47%, n=147). Most of the teachers had more than 10 years of experience (76.4%, n=291) and taught students in fourth to sixth grades (46.5%, n=177). Overall, 87.4% (n=333) of teachers had heard of ADHD, with social media being the most common source of information (39.6%, n=151). Teachers had moderate knowledge of ADHD symptoms (52.67%, n=201), but general information (29.27%, n=211) and awareness of treatment options (32.87%, n=125) were limited. Significant differences in knowledge were found based on gender, age, experience, and the type of school, with female teachers and those in private schools having higher knowledge levels. Misconceptions, particularly about treatment, were widespread, with many teachers believing in ineffective interventions like dietary changes (87.9%, n=334).

Conclusions

While teachers in the Qassim region are aware of ADHD, their overall knowledge remains insufficient, especially regarding treatment methods. Providing training programs is essential to enhance teachers' understanding and improve ADHD management in schools

## Introduction

Attention deficit hyperactivity disorder (ADHD) is a neurodevelopmental condition characterized by persistent patterns of inattention, hyperactivity, and impulsive behavior [1]. Its effects reverberate across various dimensions of a child's life, encompassing academic performance, social interactions, and emotional well-being. In the academic sphere, children with ADHD often grapple with tasks requiring sustained attention, organization, and time management, leading to potential academic underachievement and difficulties in keeping pace with standard curricula [2]. On a social level, ADHD can influence a child's interactions with peers. The traits of impulsivity and hyperactivity may pose challenges in forming and maintaining friendships, as adherence to social norms and engaging in cooperative play can be demanding for children with ADHD [3].

Emotionally, the persistent difficulties associated with ADHD may give rise to feelings of frustration and lower self-esteem in affected children. Stress stemming from struggles in both academic and social settings can contribute to an overarching sense of inadequacy. Recognizing these multifaceted effects is imperative for teachers. A lack of awareness about the condition might lead to the misinterpretation of behaviors associated with ADHD, potentially resulting in disciplinary actions rather than constructive support. Hence, understanding the common challenges faced by children with ADHD empowers teachers to implement targeted interventions and accommodate these children better, facilitating not only academic success but also positive social and emotional development [4].

Regarding the awareness levels of ADHD among teachers worldwide, a study involving 2306 teachers from nine countries (Czech Republic, Germany, Greece, Iraq, the Republic of Korea, Saudi Arabia, South Africa, United States, and Vietnam) was conducted to measure their ADHD knowledge and misconception using the Knowledge of Attention Deficit Disorders Scale (KADDS) survey. The results showed that teachers in the United States, the Czech Republic, and Germany had the highest levels of overall knowledge while those from Saudi Arabia, Vietnam, and the Republic of Korea had the lowest scores [5].

Nationally, a study performed to assess the knowledge and attitude of ADHD among male primary school teachers in Riyadh City revealed that the average level of ADHD knowledge among them was good (at least 90% of their answers were correct) [6]. Another study among primary teachers in the Albaha region found that the teachers had poor or inadequate understanding, as reflected by their incorrect answers to most of the questions [7]. Additionally, research among the female teachers at elementary schools in Jeddah showed that more than half of the teachers (54.3%) thought that they had sufficient knowledge about ADHD; however, the actual results showed a much lower level of knowledge (24.5%) [8].

To our knowledge, no previous studies have evaluated the general understanding of ADHD among teachers in the Qassim region. In light of this, we aimed to fill this gap by assessing the general knowledge of ADHD among primary school teachers in the Qassim region, Saudi Arabia.

## Materials and methods

Study design, setting, population, sample size, and sampling

This cross-sectional study was conducted among primary school teachers in the Qassim region, Saudi Arabia, from March 2024 to October 2024. A non-probability convenient sampling technique was used to select the cohort, and all primary or kindergarten teachers in the Qassim region who agreed to participate were included in the study. All the data that did not fit our criteria or those that were incomplete were excluded from the study. The sample size was calculated using the Raosoft sample size calculator application and the most recent Ministry of Education statistics for the Qassim region, Saudi Arabia; the sample size was determined to be 380 teachers with a confidence range of 95% and a sampling error of 5%.

Data collection method, management, and statistical analysis

After obtaining ethical approval, an online questionnaire was administered using Google Forms and distributed to the teachers through WhatsApp school groups in the Qassim region. The questionnaire was divided into two sections: general information and ADHD knowledge. The first section included teachers' personal information: age, gender, specialty, teaching experiences, grades taught, and type of school (International schools adopt global curricula and offer education in foreign languages, preparing students to compete in a globalized environment. Government national schools, on the other hand, follow the local curriculum and deliver instruction in Arabic, placing greater emphasis on local values and cultural heritage. Private national schools provide a blend of international and local approaches, combining elements of both systems to meet diverse educational needs). The second section assessed the teachers' understanding of ADHD using KADDS, created by Sciutto et al. [5] and translated to Arabic by Alkahtani [9]. This 36-item questionnaire covered three areas: general knowledge (15 items), symptoms and diagnosis (nine items), and treatment (12 items). Each correct answer was scored 1, while incorrect or unknown answers received a score of 0. Total scores and percentages were calculated for each participant. A perfect score would be 36, with individual section scores of 15, 9, and 12, respectively.

Statistical analysis was conducted using SPSS Statistics version 25 (IBM Corp., Armonk, NY). Categorical data were analyzed to determine frequencies and percentages. Teachers' knowledge of ADHD was assessed across three domains: general knowledge, symptoms/diagnosis, and treatment. The total scores of these domains were calculated, and comparisons of mean scores were made concerning the demographic data of the teacher participants. Given the non-normal distribution of knowledge scores (p<0.001) revealed by the Shapiro-Wilk test, nonparametric tests were employed. The Mann-Whitney U test was used for two-group comparisons, while the Kruskal-Wallis test was applied for multiple-group comparisons. A p-value <0.05 was considered statistically significant.

Ethical consideration

Ethical approval was obtained from the Research Ethics Committee, Deanship of Scientific Research, Qassim University, Saudi Arabia (approval no: 24-82-21). All the collected data were kept confidential. Only the researchers had access to participant data. This study presents only a summary of the statistics and does not use personal information. Written consent was obtained from all participants after they were provided a brief description of the study, its purpose, and their right to withdraw from participation at any time.

## Results

Table 1 provides a demographic overview of the 381 teachers participating in the study. In terms of gender, the sample was predominantly female (58.8%, n=224) with 41.2% (n=157) being male. The majority of participants were in their 40s (47%, n=147), followed by those in their 30s (31.8%, n=121). Most teachers were employed in public schools (93.2%, n=355), and the majority had more than 10 years of teaching experience (76.4%, n=291). The majority of teachers were instructing students in the fourth to sixth grades (n=177, 46.5%), followed by the first to third grades (n=140, 36.7%). Most teachers were working with girls-only classes (n=165, 43.3%) and boys-only classes (n=166, 43.6%), followed by mixed-gender classes (n=50, 13.1%). A high proportion of participants (n=333, 87.4%) were familiar with ADHD, with social media being the most common source of information (n=151, 39.6%).

**Table 1:** General characteristics of the participants (n=381) ADHD: attention deficit hyperactivity disorder

Characteristics	N	%
Gender		
Female	224	58.8
Male	157	41.2
Age group, years		
20-30	30	7.9
31-40	121	31.8
41-50	179	47.0
51-60	51	13.4
Type of school		
International	2	0.5
Private	24	6.3
Public	355	93.2
Student's gender		
Girl students only	165	43.3
Girls and boys	50	13.1
Boy students only	166	43.6
Grades taught		
1st-3rd	140	36.7
4th–6th	177	46.5
Kindergarten	64	16.8
Teaching experience, years		
5-10	46	12.1
Less than 5	44	11.5
More than 10	291	76.4
Have you heard about ADHD?		
No	48	12.6
Yes	333	87.4
If yes, what is your source of information?		
I do not know	43	11.3
Know someone with ADHD	94	24.7
From books, newspapers	40	10.5
Medical websites	35	9.2
Social media	151	39.6

**Figure 1:**
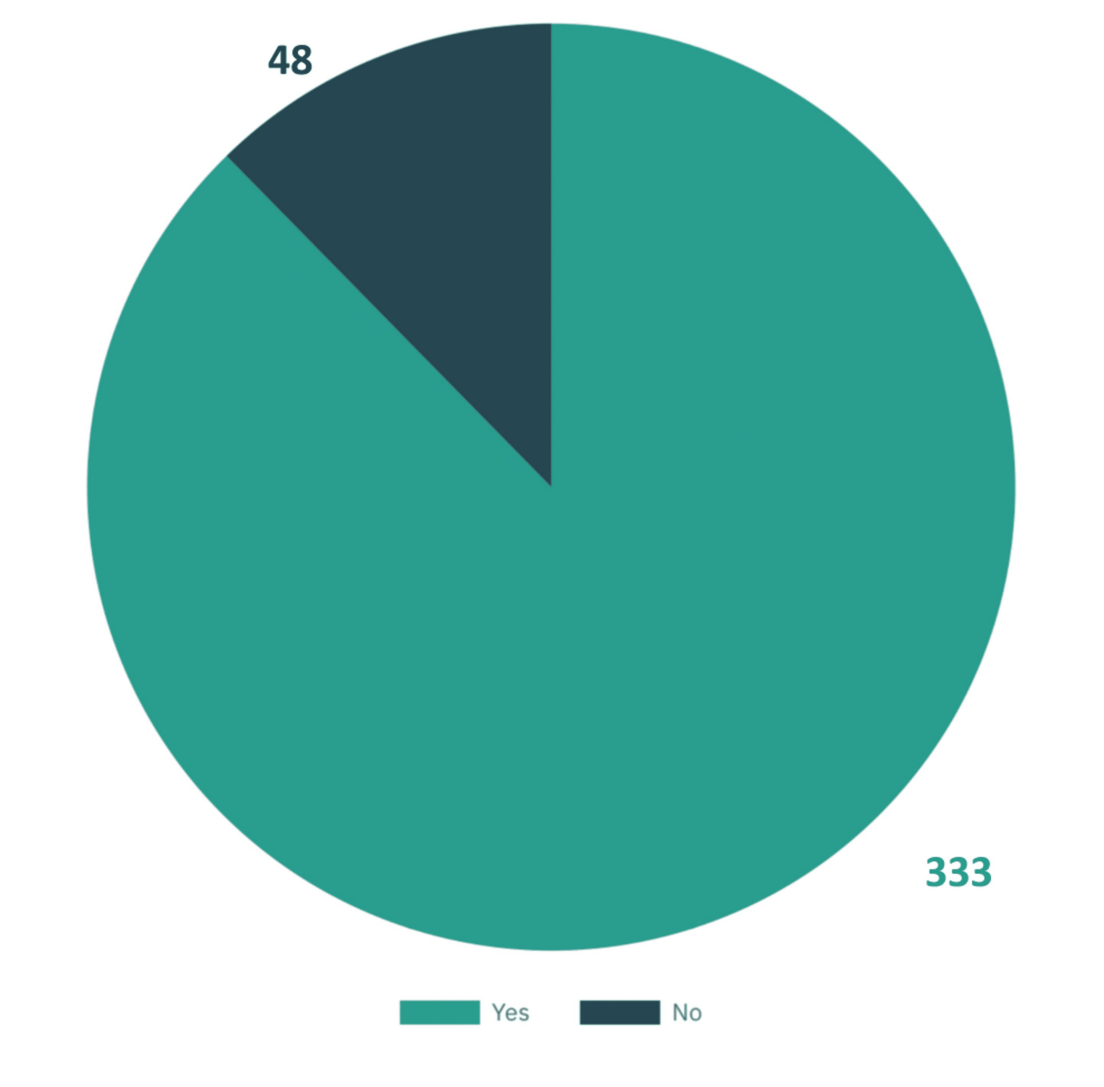
Have you heard about ADHD? ADHD: attention deficit hyperactivity disorder

**Figure 2:**
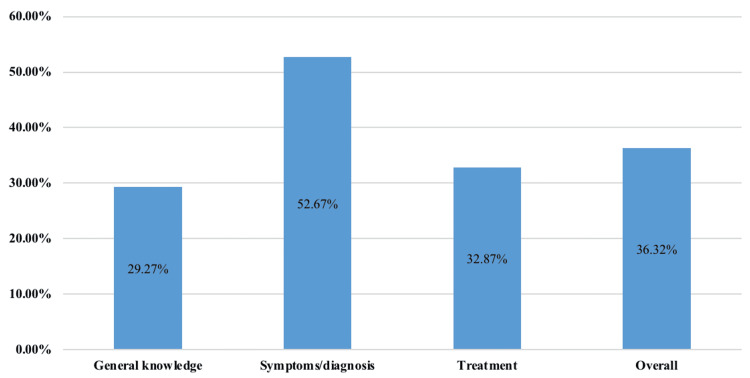
Proportion of participants by levels of ADHD-related knowledge ADHD: attention deficit hyperactivity disorder

As depicted in Figure 1, 87% (n=333) of participants had heard about ADHD, while only 13% (n=48) had not. This indicates that ADHD is a relatively well-known condition, with a significant portion of the population being aware of it. As shown in Figure 2, primary school teachers had a relatively good understanding of ADHD symptoms and diagnosis, but their level of general information and awareness of treatment options were limited. While 52.67% (n=200) of teachers had a good grasp of symptoms and diagnosis, only 29.27% (n=111) had good general knowledge, and 32.87% (n=125) had a good understanding of treatment. Overall, 36.32% (n=137) of teachers had a good overall understanding of ADHD.

Table 2 provides a detailed overview of the teachers' knowledge regarding ADHD. The results indicate that while teachers generally had a good understanding of some core concepts related to ADHD, there are still significant gaps in their knowledge. For example, a significant number of teachers were unaware that ADHD can be diagnosed in adults (n=211, 55.4%) or that symptoms of depression are more common in ADHD children (n=213, 55.9%). Additionally, many teachers incorrectly believed that ADHD children are more likely to experience problems in novel situations than in familiar ones (n=334, 87.7%) and that there are specific physical features that can be used to diagnose ADHD (n=323, 84.8%). However, the teachers demonstrated a strong understanding of the link between ADHD and poor school performance (n=222, 58.3%) and the potential influence of chaotic home environments on ADHD symptoms (n=170, 44.6%). 

**Table 2:** General information regarding ADHD among teachers ADHD: attention deficit hyperactivity disorder

General knowledge	Right answer	
	N	%
Most estimates suggest that ADHD occurs in approximately 15% of school-age children. (False)	32	8.4
ADHD children are typically more compliant with their fathers than with their mothers. (True)	123	32.3
ADHD is more common in the 1st degree biological relatives (i.e., mother and father) of children with ADHD than in the general population. (True)	111	29.1
It is possible for an adult to be diagnosed with ADHD. (True)	170	44.6
Symptoms of depression are found more frequently in ADHD children than in non‑ADHD children. (True)	168	44.1
Most ADHD children “outgrow” their symptoms by the onset of puberty and subsequently function normally in adulthood. (False)	44	11.5
If an ADHD child is able to demonstrate sustained attention to video games or TV for over an hour, that child is also able to sustain attention for at least an hour of class or homework. (False)	81	21.3
A diagnosis of ADHD by itself makes a child eligible for placement in special education. (False)	37	9.7
ADHD children generally experience more problems in novel situations than in familiar situations. (False)	47	12.3
There are specific physical features that can be identified by medical doctors (e.g., pediatricians) in making a definitive diagnosis of ADHD. (False)	58	15.2
In school-age children, the prevalence of ADHD in males and females is equivalent. (False)	100	26.2
In very young children (less than 4 years of age), the problem behaviors of ADHD children (e.g., hyperactivity, inattention) are distinctly different from age‑appropriate behaviors of non‑ADHD children. (False)	36	9.4
Children with ADHD are more distinguishable than normal children in a classroom setting than in a free play situation. (True)	274	71.9
The majority of ADHD children evidence some degree of poor school performance in elementary school years. (True)	222	58.3
Symptoms of ADHD are often seen in non‑ADHD children who come from inadequate and chaotic home environments. (True)	170	44.6

Table 3 provides a detailed overview of the teachers' knowledge regarding ADHD symptoms and diagnosis. The results indicate that teachers generally had a good understanding of the core symptoms of ADHD and the diagnostic criteria. For example, a high proportion of teachers correctly identified that ADHD children are often distracted by extraneous stimuli (n=308, 80.8%), fidget or squirm in their seats (n=308, 80.8%), and have difficulties organizing tasks and activities (n=246, 64.6%). Additionally, most teachers were aware that a diagnosis of ADHD requires the presence of symptoms before age seven (n=187, 49.1%) and in two or more settings (n=261, 68.5%). However, there were still some areas where teachers had misconceptions, such as believing that ADHD children often have an inflated sense of self-esteem (n=72, 18.9%) or a history of stealing and destroying others' things (n=79, 20.7%).

**Table 3:** Knowledge of teachers regarding ADHD symptoms and diagnosis ADHD: attention deficit hyperactivity disorder

Symptoms/diagnosis	Right answer	
	N	%
ADHD children are frequently distracted by extraneous stimuli. (True)	308	80.8
In order to be diagnosed with ADHD, the child’s symptoms must have been present before age 7. (True)	187	49.1
One symptom of ADHD children is that they have been physically cruel to other people. (False)	103	27
ADHD children often fidget or squirm in their seats. (True)	308	80.8
It is common for ADHD children to have an inflated sense of self‑esteem or grandiosity. (False)	72	18.9
ADHD children often have a history of stealing or destroying other people’s things. (False)	79	20.7
Current wisdom about ADHD suggests two clusters of symptoms: one of inattention and another consisting of hyperactivity/impulsivity. (True)	242	63.5
In order to be diagnosed with ADHD, a child must exhibit relevant symptoms in two or more settings (e.g., home and school). (True)	261	68.5
ADHD children often have difficulties organizing tasks and activities. (True)	246	64.6

Table 4 presents a summary of teachers' knowledge regarding ADHD treatment, with each statement accompanied by the percentage of correct responses and the corresponding number.

**Table 4:** Knowledge of teachers regarding ADHD treatment ADHD: attention deficit hyperactivity disorder

Treatment	Right answer	
	N	%
Current research suggests that ADHD is largely the result of ineffective parenting skills. (False)	177	46.5
Antidepressant drugs have been effective in reducing symptoms for many ADHD children. (True)	116	30.4
Parent and teacher training in managing an ADHD child is generally effective when combined with medication treatment. (True)	290	76.1
When treatment of an ADHD child is terminated, it is rare for the child’s symptoms to return. (False)	82	21.5
Side effects of stimulant drugs used for the treatment of ADHD may include mild insomnia and appetite reduction. (True)	142	37.3
Individual psychotherapy is usually sufficient for the treatment of most ADHD children. (False)	97	25.5
In severe cases of ADHD, medication is often used before other behavior modification techniques are attempted. (True)	171	44.9
Reducing dietary intake of sugar or food additives is generally effective in reducing the symptoms of ADHD. (False)	46	12.1
Stimulant drugs are the most common type of drug used to treat children with ADHD. (True)	92	24.1
Behavioral/psychological interventions for children with ADHD focus primarily on the child’s problems with inattention. (False)	41	10.8
Electroconvulsive therapy (i.e., shock treatment) has been found to be an effective treatment for severe cases of ADHD. (False)	65	17.1
Treatments for ADHD which focus primarily on punishment have been found to be the most effective in reducing the symptoms of ADHD. (False)	184	48.3

Teachers frequently held misconceptions about ADHD treatment. For instance, only 30.4% (n=116) were aware of the effectiveness of antidepressant drugs, while 78.5% (n=299) incorrectly believed that symptoms rarely return after treatment termination, and only 21.5% (n=82) disagreed. Furthermore, 74.5% (n=284) believed that individual psychotherapy is sufficient for most ADHD children, while only 25.5% (n=97) accurately recognized its limitations. Additionally, 87.9% (n=335) believed that reducing sugar intake was an effective intervention, while only 12.1% (n=46) recognized its ineffectiveness. Similarly, 82.9% (n=316) believed that electroconvulsive therapy was a viable treatment option, despite only 17.1% (n=65) being correct. Finally, 89.2% (n=340) incorrectly believed that behavioral/psychological interventions primarily focus on inattention, while only 10.8% (n=41) disagreed. Notably, only 24.1% (n=92) were aware that stimulant drugs are the most common type of medication used to treat ADHD in children.

Teachers demonstrated a good understanding of certain aspects of ADHD treatment. For instance, 76.1% (n=290) correctly identified the effectiveness of combined medication and parent/teacher training, while 48.3% (n=184) accurately recognized the ineffectiveness of punishment-focused treatments. Additionally, 46.5%(n=177) correctly identified that ADHD is not primarily caused by ineffective parenting. However, only 37.3% (n=142) were aware of the potential side effects of stimulant drugs. Furthermore, 44.9% (n=171) accurately recognized the importance of medication in severe cases of ADHD, often used before other behavioral modification techniques.

Teachers’ knowledge regarding various aspects of attention deficit hyperactivity disorder

General Information

The results of the Kruskal-Wallis H and Mann-Whitney U tests revealed that several factors were significantly associated with teachers' general information about ADHD. These factors included gender, type of school, awareness of ADHD, and source of information. Female teachers demonstrated significantly higher levels of knowledge compared to male teachers (mean rank=207.04, p=0.016). Teachers from international or private schools exhibited a greater depth of knowledge compared to those from public schools (mean ranks=258.35 and 186.07, respectively, p<0.001). Additionally, teachers who had heard about ADHD and obtained information from sources like books or newspapers displayed significantly higher levels of general knowledge compared to those who were unaware of ADHD or relied on medical websites or social media (Table 5).

**Table 5:** Factors associated with teachers' knowledge regarding ADHD ^a^Kruskal-Wallis H. ^b^Mann-Whitney U. ^*^Statistically significant ADHD: attention deficit hyperactivity disorder

	ADHD knowledge score								
	General knowledge			Symptoms/diagnosis			Treatment		
	P-value	Test value	Mean rank	P-value	Test value	Mean rank	P-value	Test value	Mean rank
Gender									
Female	0.016^*^	15066^b^	179.76	0.194	16226^b^	197.06	0.72	17208^b^	189.32
Male			207.04			182.35			193.39
Age group, years									
20-30	0.366	3.174^a^	215.4	0.012^*^	11.026^a^	158	0.896	0.603^a^	198.93
31-40			197.1			171.33			194.87
41-50			181.87			207.03			189.2
51-60			194.25			200.82			183.45
Type of school									
International private	0.001^*^	2864^b^	258.35	0.46	4219.5^b^	175.79	<0.000	2674^b^	265.65
Public			186.07			192.11			185.53
Student's gender									
Girl students only	0.263	2.674^a^	181.22	0.808	0.426^a^	192.33	0.566	1.140^a^	197.66
Girls and boys			190.63			181.65			182.7
boy students only			200.84			192.49			186.88
Grades taught									
1st-3rd	0.15	3.790^a^	201.27	0.328	2.232^a^	180.09	0.007^*^	9.826^a^	211.42
4th-6th			179.31			197.64			173.04
Kindergarten			200.87			196.52			196.02
Teaching experience, years									
5-10	0.364	2.020^a^	192.65	<0.000^*^	19.750^a^	133.14	0.197	3.249^a^	212.47
Less than			212.51			164.97			205.03
More than 10			187.49			204.08			185.48
Have you heard about ADHD?									
No	<0.000^*^	5367.5^b^	136.32	<0.000^*^	5415^b^	137.31	<0.000^*^	4789^b^	124.27
Yes			198.88			198.74			200.62
If yes, what is your source of information?									
I do not know	<0.000^*^	30.964^a^	113.49	0.001^*^	17.901^a^	132.86	<0.000^*^	31.159^a^	99.69
Know someone with ADHD			200.86			210.35			195.43
From books or newspaper			217.29			164.2			193.59
Medical websites			214.66			184.23			203.51
Social media			172.85			182.54			189.03

Symptoms and Diagnosis

The results in Table 5 show that several factors were significantly associated with teachers' knowledge regarding symptoms/diagnosis of ADHD. These factors included age, teaching experience, awareness of ADHD, and source of information. The highest scores for knowledge of symptoms/diagnosis were reported among teachers aged 41-50 years and 51-60 years (mean rank=207.03 for both groups), whereas the lowest score was reported among teachers aged 20-30 years (mean rank=158, p=0.012). Additionally, teachers with more than 10 years of teaching experience demonstrated significantly higher knowledge of symptoms/diagnosis compared to those with 5-10 years of experience (mean ranks=204.08 and 133.14, respectively, p<0.000). Teachers who had heard about ADHD had significantly higher mean ranks for knowledge of symptoms/diagnosis compared to those who had not (mean ranks=198.74, p<0.000). Teachers who learned about ADHD from books or newspapers had significantly higher mean ranks for knowledge of symptoms/diagnosis compared to those who learned from social media (p<0.000).

Significant Findings (P<0.05)

Age: Teachers in the 20-30 age group had significantly lower mean ranks for knowledge of symptoms/diagnosis compared to teachers in the 41-50 and 51-60 age groups. Specifically, the mean rank for teachers aged 20-30 was 158, while the mean ranks for teachers aged 41-50 and 51-60 were 207.03 and 207.03, respectively.

Teaching experience: Teachers with 5-10 years of experience had significantly lower mean ranks for knowledge of symptoms/diagnosis compared to teachers with less than five years of experience. The mean rank for teachers with 5-10 years of experience was 133.14, while the mean rank for teachers with less than five years of experience was 204.08.

Awareness of ADHD: Teachers who had heard about ADHD had significantly higher mean ranks for knowledge of symptoms/diagnosis compared to teachers who had not. The mean rank for teachers who had heard about ADHD was 137.31, while the mean rank for teachers who had not heard about ADHD was 198.74.

Source of information: Teachers who had learned about ADHD from books or newspapers had significantly higher mean ranks for knowledge of symptoms/diagnosis compared to those who had learned from medical websites or social media. The mean rank for teachers who had learned from books or newspapers was 184.23, while the mean rank for teachers who had learned from medical websites or social media was 182.54

Treatment

The findings suggest that factors such as type of school, grades taught, awareness of ADHD, and source of information can influence teachers' knowledge of treatment options for ADHD. Teachers from international private schools demonstrated significantly higher knowledge levels compared to public school teachers (mean ranks=265.65 and 185.53, respectively, p<0.000). Teachers who taught first-third grades exhibited higher knowledge compared to those who taught kindergarten (mean ranks=173.04 and 179.31, p=0.007). Additionally, teachers who had heard about ADHD and obtained information from books or newspapers displayed significantly higher knowledge levels compared to those who were unaware of ADHD or relied on medical websites or social media (mean ranks for awareness: 124.27 vs. 200.62, p<0.000; mean ranks for source of information: 203.51 vs. 189.03, p<0.000).

Significant Findings (P<0.05)

Type of school: Teachers from international private schools had significantly higher mean ranks for knowledge of treatment compared to public school teachers. Specifically, the mean rank for teachers from international private schools was 265.65, while the mean rank for teachers from public schools was 185.53 (p<0.000).

Grades taught: Teachers who taught first-third grades had significantly higher mean ranks for knowledge of treatment compared to teachers who taught kindergarten. The mean rank for teachers who taught first-third grades was 173.04, while the mean rank for teachers who taught kindergarten was 179.31 (p=0.007).

Awareness of ADHD: Teachers who had heard about ADHD had significantly higher mean ranks for knowledge of treatment compared to teachers who had not. The mean rank for teachers who had heard about ADHD was 124.27, while the mean rank for teachers who had not heard about ADHD was 200.62 (p<0.000).

Source of information: Teachers who had learned about ADHD from books or newspapers had significantly higher mean ranks for knowledge of treatment compared to those who had learned from medical websites or social media. The mean rank for teachers who had learned from books or newspapers was 203.51, while the mean rank for teachers who had learned from medical websites or social media was 189.03 (p<0.000).

**Figure 3:**
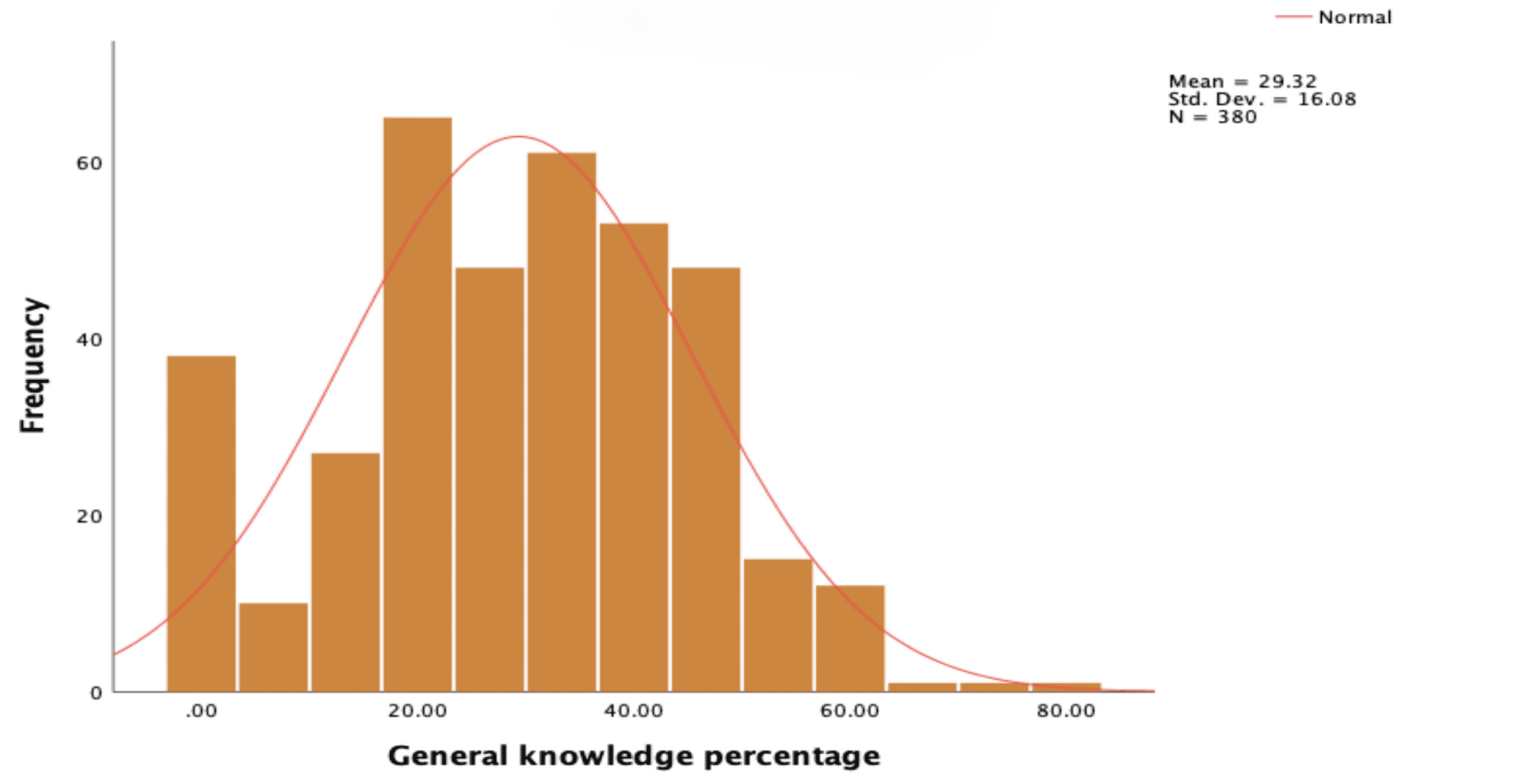
Distribution of the level of knowledge regarding ADHD-related general information ADHD: attention deficit hyperactivity disorder

Figure 3 shows the distribution of the level of general knowledge among the participants regarding ADHD. The average value was 29.32%, with a standard deviation of 16.08. This indicates a relatively low level of understanding, with a significant amount of variation among individuals. Some had very low knowledge, while others had high knowledge, although the majority fell within the lower to moderate range.

**Figure 4:**
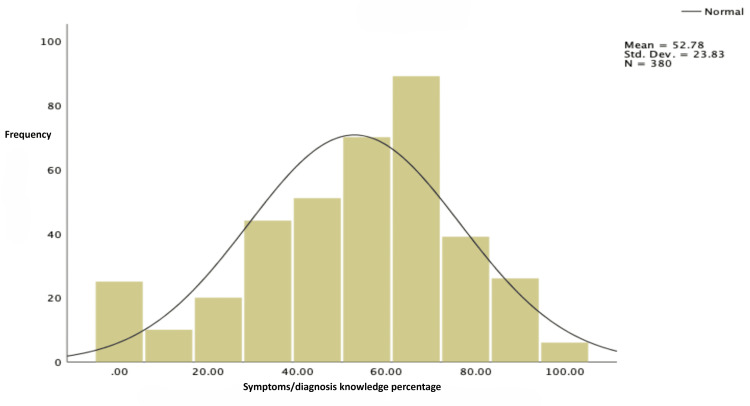
Level of knowledge regarding symptoms/diagnosis ADHD: attention deficit hyperactivity disorder

Figure 4 shows the distribution of knowledge levels among the participants regarding ADHD symptoms and diagnosis. The average value was 52.78%, with a standard deviation of 23.83. This indicates a moderate level of understanding but with significant variation among individuals, ranging from very low to high knowledge levels.

**Figure 5:**
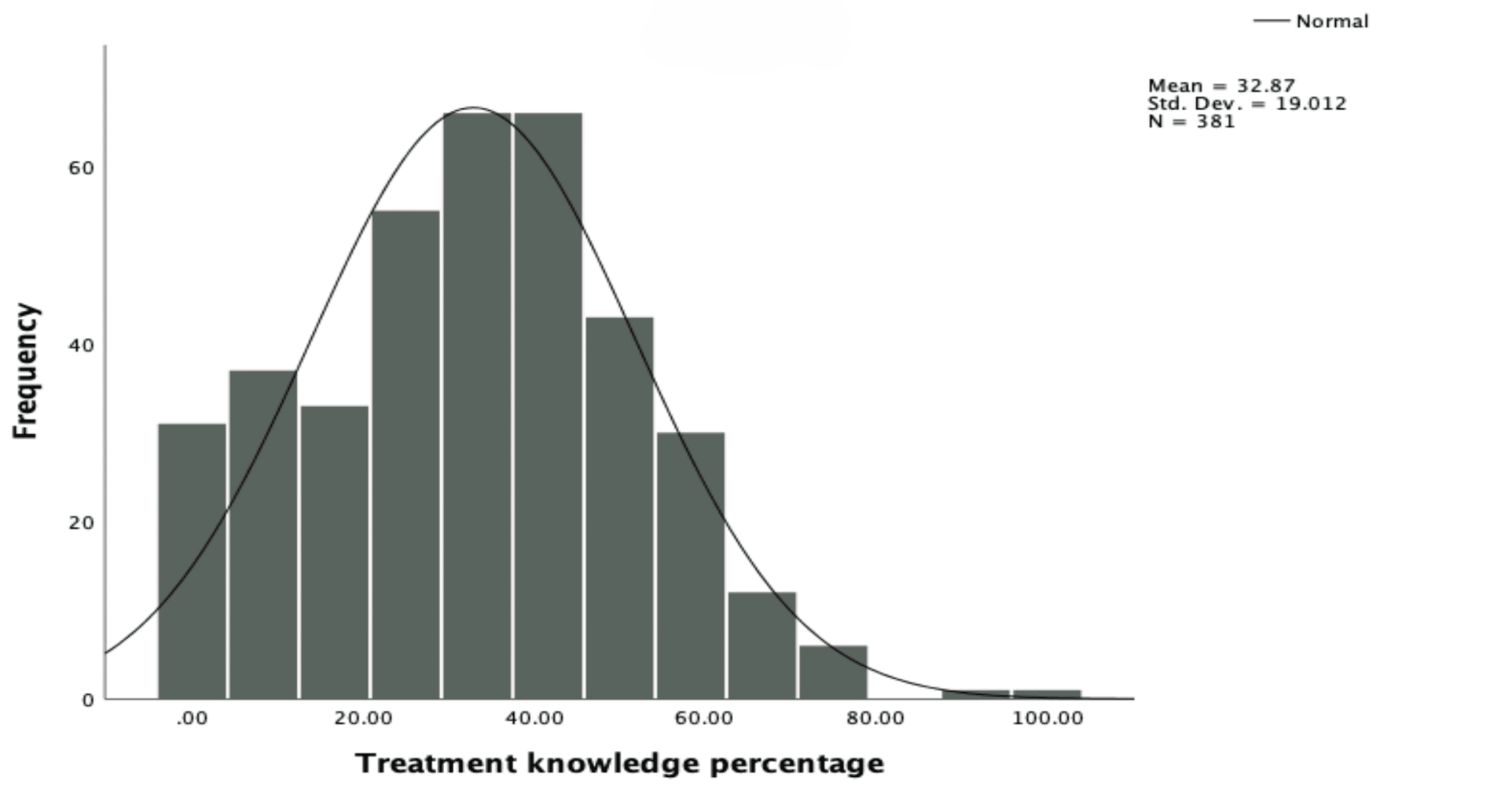
Distribution of the level of knowledge regarding ADHD treatment ADHD: attention deficit hyperactivity disorder

Figure 5 shows the distribution of levels of treatment-related knowledge among the participants. The average treatment knowledge value was 32.87%, with a standard deviation of 19.012. This indicates that while the average knowledge was moderate, there was a wide range of scores, suggesting that some individuals had very low knowledge, while others had high knowledge.

**Figure 6:**
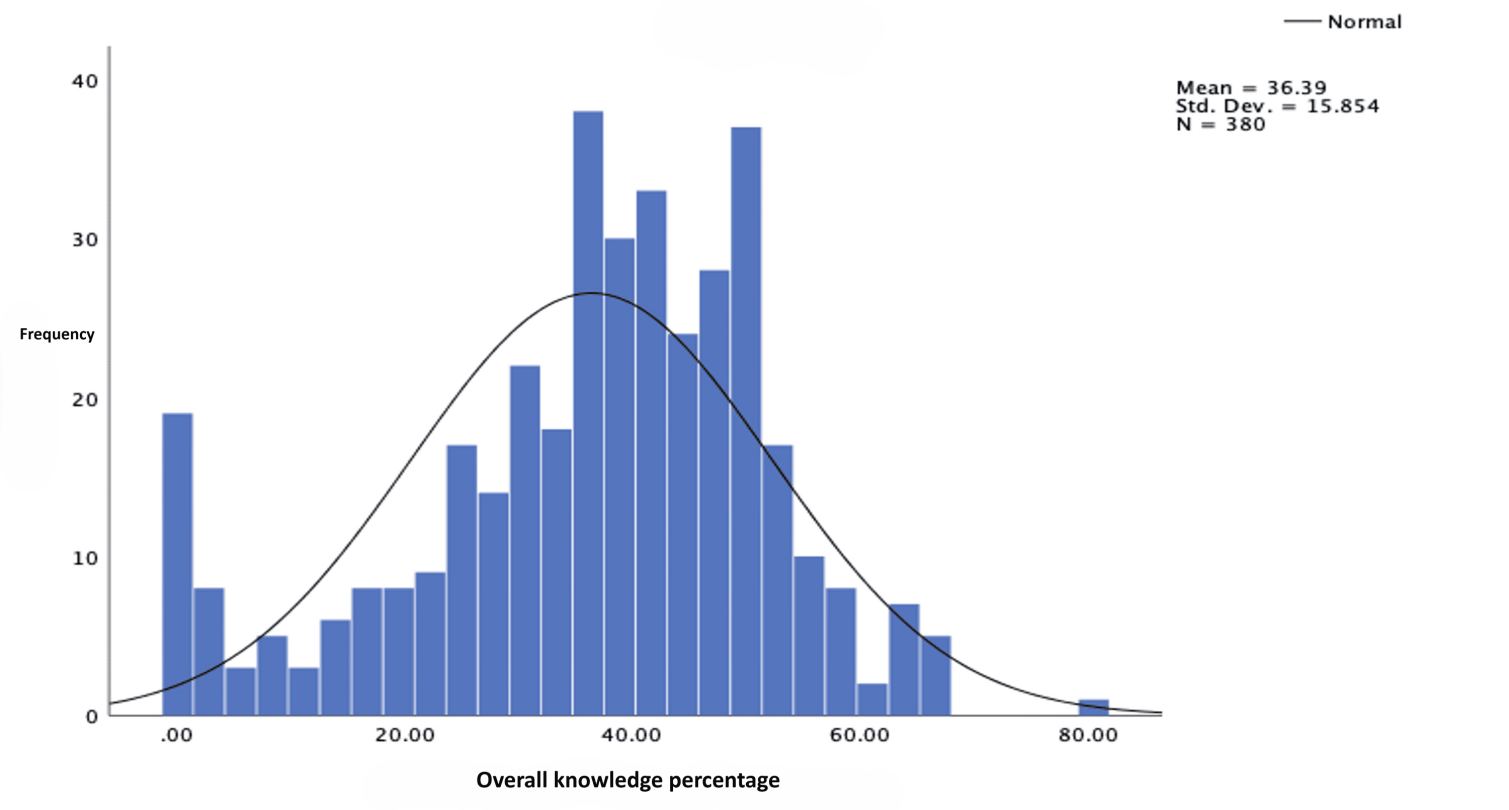
Distribution of the level of overall knowledge regarding ADHD ADHD: attention deficit hyperactivity disorder

As illustrated in Figure 6, the overall average knowledge score about ADHD was only 36.39%, which is considered low. Additionally, the standard deviation of 15.854 indicates a wide range of scores, suggesting that some individuals had very low levels of knowledge while others had relatively high levels. This information is based on the data presented in Figure 2 and Figure 6.

## Discussion

This study explores the knowledge and misconceptions among primary school teachers in the Qassim region, Saudi Arabia about ADHD, focusing on the teachers' overall understanding of the disorder. Teachers demonstrated moderate knowledge about ADHD symptoms and diagnosis but had limited understanding in terms of general awareness and treatment options. While 52.67% (n=200) of the teachers had a good grasp of ADHD symptoms, only 29.27% (n=111) demonstrated adequate general knowledge. This finding aligns with a multi-country study where teachers’ knowledge of ADHD was generally insufficient [9], emphasizing the importance of adequate ADHD-related training and experience to improve awareness [10].

The gender and age of teachers were significant factors affecting their knowledge of ADHD, with female teachers and those aged 41-50 years showing higher levels of understanding, particularly regarding symptoms and diagnosis. These findings are consistent with a study conducted in Jeddah, where older teachers showed significantly better knowledge of ADHD. In that study, despite more than half of the teachers believing they had knowledge of ADHD, only 24.5% actually demonstrated adequate understanding [8]. This suggests that misconceptions persist despite perceived awareness, highlighting the need for more targeted training programs for educators.

Teachers' knowledge of ADHD treatment was notably poor, with significant misconceptions, such as the belief that dietary changes or electroconvulsive therapy could be effective treatments. Only 24.1% (n=91) of teachers correctly identified stimulant drugs as the most common treatment for ADHD. Similar results were found in a study from Albaha, where most teachers displayed inadequate knowledge about ADHD management [7]. This information gap emphasizes the importance of enhancing teacher training to provide accurate knowledge of effective interventions [11].

Teachers with more than 10 years of experience showed significantly higher knowledge in diagnosing ADHD symptoms compared to those with fewer years of teaching. This mirrors findings from a study conducted in Medina, which also reported that more experienced teachers had better knowledge of ADHD symptoms and treatments [12]. However, both studies reveal that, overall, teachers' knowledge remains suboptimal, especially regarding treatment. This emphasizes the need for continuous professional development, particularly in managing ADHD in the classroom.

Teachers who learned about ADHD from books or newspapers had significantly higher levels of knowledge compared to those relying on social media or medical websites. This is supported by the findings in Riyadh, where teachers who read books or attended courses had a better understanding of ADHD than those who did not [6]. This highlights the importance of providing teachers with credible, evidence-based resources to improve their understanding of the disorder. The relationship between teachers' knowledge and their source of information is critical. Social media was the most common source of information, yet those who relied on it had lower levels of accurate knowledge. This finding is supported by the multi-country study, which highlighted the need for structured training programs to improve teachers' confidence and knowledge about ADHD [9]. This suggests that unverified sources may contribute to the spread of misconceptions, further stressing the need for reliable educational materials.

Another significant finding involves the influence of the type of school on teachers’ knowledge, with teachers from international and private schools demonstrating significantly higher knowledge than those from public schools. This finding is consistent with another study, where teachers from more resource-rich environments, such as private schools, had better access to training and resources [13]. This discrepancy underscores the need for equitable access to ADHD training across all school types to ensure that all students receive adequate support [14].

Teachers who had prior experience with ADHD, either through personal connections or professional encounters, showed significantly better understanding of both symptoms and treatments. This mirrors findings from one study, where teachers with experience of teaching students with ADHD had higher knowledge scores [15]. This reinforces the idea that direct exposure to ADHD, coupled with adequate training, plays a crucial role in enhancing teachers' knowledge.

A notable knowledge gap was identified in ADHD treatment, particularly with misconceptions about the role of psychotherapy and dietary interventions. Many teachers incorrectly believed that reducing sugar intake could effectively manage ADHD symptoms. This finding is consistent with the findings of a study from Makkah City, where teachers also showed an inadequate understanding of ADHD treatment [16]. These persistent misconceptions call for comprehensive training that emphasizes evidence-based treatment approaches, such as behavioral interventions and medication management.

This study has a few limitations. The sample size was limited to primary school teachers in the Qassim region, and hence the findings may not be representative of teachers in other regions. Moreover, this study relied on self-reported data, which may have led to biases or inaccurate responses. The knowledge assessment tool focused only on general knowledge, symptoms, and treatment, without exploring other important aspects such as the social and emotional impacts of ADHD. The study was cross-sectional in design, and it could not track changes in teachers' knowledge over time.

Future research should include a larger and more diverse sample of teachers from different regions to better understand ADHD knowledge on a national level. Longitudinal studies would be helpful to assess how teachers' knowledge and attitudes toward ADHD evolve over time, especially after receiving training. Future studies should also explore other important factors, such as teachers' classroom strategies for managing ADHD and their perceptions of the emotional and social impacts of the disorder on students. It would be valuable to assess the effectiveness of various teacher training programs aimed at improving ADHD awareness and management skills.

## Conclusions

While primary school teachers in the Qassim region have a moderate understanding of ADHD, particularly in recognizing its symptoms, they lack sufficient knowledge about its general aspects and treatment options. Female teachers, older teachers, and those with more experience generally had better knowledge. However, many misconceptions still exist, especially regarding treatment methods, with some teachers believing in ineffective interventions like dietary changes. While many teachers are aware of ADHD, their understanding is limited, particularly in managing the condition. This study highlights the urgent need for training programs to provide teachers with accurate information and better equip them to support students with ADHD.
